# Multi-Channel Microscale Nerve Cuffs for Spatially Selective Neuromodulation

**DOI:** 10.3390/mi15081036

**Published:** 2024-08-15

**Authors:** Morgan Riley, FNU Tala, Katherine J. Johnson, Benjamin C. Johnson

**Affiliations:** 1 Biomedical Engineering Doctoral Program, Boise State University, Boise, ID 83725, USA; 2 Department of Electrical and Computer Engineering, Boise State University, Boise, ID 83725, USA; 3 Office of Research Compliance, Boise State University, Boise, ID 83725, USA

**Keywords:** selective, neuromodulation, micro-scale, multi-channel, cuff, custom

## Abstract

Peripheral nerve modulation via electrical stimulation shows promise for treating several diseases, but current approaches lack selectivity, leading to side effects. Exploring selective neuromodulation with commercially available nerve cuffs is impractical due to their high cost and limited spatial resolution. While custom cuffs reported in the literature achieve high spatial resolutions, they require specialized microfabrication equipment and significant effort to produce even a single design. This inability to rapidly and cost-effectively prototype novel cuff designs impedes research into selective neuromodulation therapies in acute studies. To address this, we developed a reproducible method to easily create multi-channel epineural nerve cuffs for selective fascicular neuromodulation. Leveraging commercial flexible printed circuit (FPC) technology, we created cuffs with high spatial resolution (50 μm) and customizable parameters like electrode size, channel count, and cuff diameter. We designed cuffs to accommodate adult mouse or rat sciatic nerves (300–1500 μm diameter). We coated the electrodes with PEDOT:PSS to improve the charge injection capacity. We demonstrated selective neuromodulation in both rats and mice, achieving preferential activation of the tibialis anterior (TA) and lateral gastrocnemius (LG) muscles. Selectivity was confirmed through micro-computed tomography (*μ*CT) and quantified through a selectivity index. These results demonstrate the potential of this fabrication method for enabling selective neuromodulation studies while significantly reducing production time and costs compared to traditional approaches.

## 1. Introduction

**Figure 1:**
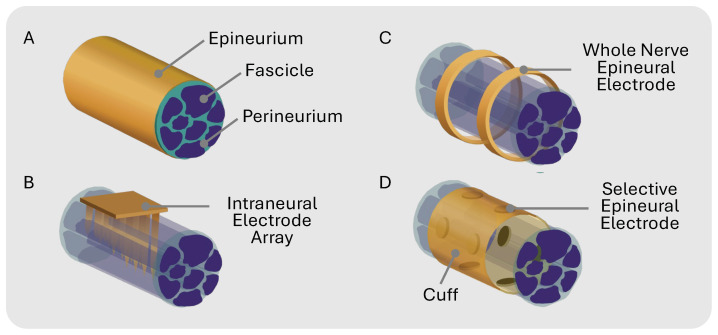
(**A**) Peripheral nerve general anatomy. (**B**) Intraneural electrode array that penetrates epineurium and specific fascicles for selective stimulation. (**C**) Multi-channel electrodes that unspecifically stimulate whole nerve epineurally. (**D**) Epineural electrodes in cuff for selective stimulation.

Peripheral nerve modulation with electrical stimulation has emerged as a therapeutic approach for a wide range of physiological and neurological disorders [1,2] such as depression, epilepsy, incontinence, rheumatoid arthritis, and chronic pain [3,4,5,6]. Traditional therapies use cuff electrodes to chronically interface with the target nerve. Cuffs with circumpolar electrodes where the contacts wrap all the way around a nerve lack the precision required to isolate individual nerve fibers, leading to non-specific stimulation (Figure 1C). This whole-nerve stimulation can cause off-target effects such as muscle twitches or pain in innervated regions [7]. Additionally, non-selective nerve stimulation may limit the therapeutic window since overall stimulation must be reduced to compensate for unwanted side effects.

Selectively activating a single fascicle within a fascicle-dense nerve is exceptionally challenging for multiple reasons: (1) the fascicle topology is unknown prior to histological analysis because nerve size and branching patterns can vary between individuals [8]; (2) stimulation intensity varies with distance, where fascicles deeper in the nerve receive less stimulation than fascicles closer to the electrode interface [9,10]; and (3) the stimulation threshold varies with fiber diameter, where larger-diameter fibers have a lower threshold than smaller-diameter fibers [11]. Prior efforts, such as the Utah Slant Electrode Array and transverse intrafascicular multi-channel electrode (TIME) [12,13], use penetrating intraneural electrodes to improve selectivity (Figure 1B). While more invasive than cuff electrodes, penetrating electrodes enhance stimulation selectivity by placing the electrodes in closer contact with the fascicles [14]. The selectivity of non-penetrating epineural cuffs can be improved by using a flat interface nerve electrode (FINE) that compresses the nerve, forcing the fascicles to spread out and maximize the surface area for targeted stimulation [14,15,16,17]. Multi-contact cuffs have the potential to enable the recording and stimulation of individual nerve fascicles within complex nerves innervating multiple targets (Figure 1D). Spatially selective stimulation with multicontact epineural cuffs can improve therapeutic results while mitigating off-target side effects in bioelectronic therapies [3,18,19].

For pre-clinical, selective neuromodulation pilot studies, multi-channel commercial cuffs are costly and have limited customization options, while building custom solutions is technically challenging. Researchers typically rely on advanced microfabrication techniques (e.g., photolithography) to manufacture multi-contact nerve cuffs for peripheral neuromodulation, which offer exceptional spatial resolution and material versatility [1,20]. In contrast, additive manufacturing techniques have emerged as an intriguing alternative for rapidly prototyping multi-contact cuffs tailored for acute investigations, despite their relatively lower spatial resolution compared to photolithography [21,22]. However, both photolithography and additive manufacturing require the expertise of skilled researchers and technicians, along with access to specialized equipment, which can drastically slow design iterations because of the required training, intricate parametrization challenges, and substantial costs. Researchers looking to test their designs without the expense and equipment often manually assemble the cuffs [23,24]. This method of manual fabrication also has limitations, such as compromised spatial resolution, increased product variability, and prolonged assembly duration.

**Figure 2:**
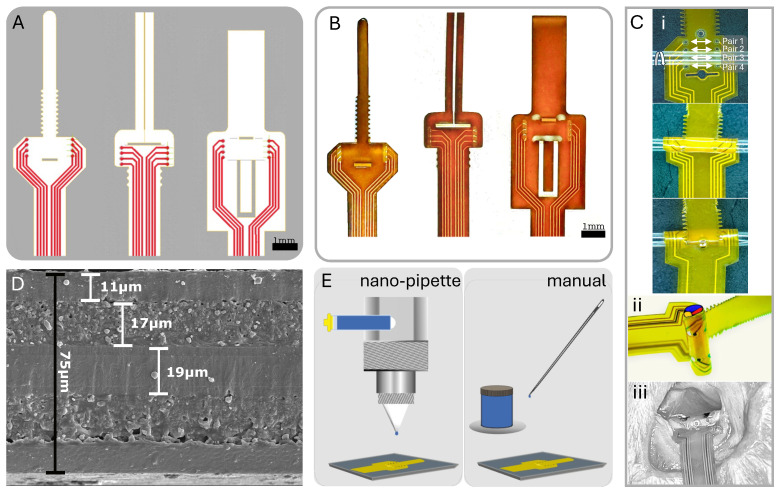
Overview of cuff design and post-fabrication electrode coating methods. (**A**) CAD design of three unique cuff designs. (**B**) Physical cuff produced from FPC fabrication. (**C**) (**i**) Zip-tie design wrapping with longitudinal pairs noted; (**ii**) 3D nerve wrap simulation on tubing; (**iii**) in vivo view of cuff on rat sciatic nerve. (**D**) SEM of polyimide substrate with layer thicknesses determined. (**E**) Post-fabrication PEDOT:PSS application to electrodes via nano-pipette and manual application.

Each of the fabrication methods in Table 1 has its merits, but their common drawbacks—time-intensive production, high non-recurring engineering (NRE) costs, and challenging design iteration—should be considered when selecting a method. To address these issues, we propose a cost-effective and time-effective solution using commercial flexible printed circuit (FPC) technology in which the cuffs can be custom designed with a spatial resolution of 50 μm and require minimal post-processing. These cuffs can be easily manufactured without specialized cleanroom facilities or labor-intensive manual assembly processes. In this work, we designed a multi-contact nerve cuff prototype with high stimulation capacity for targeted neuromodulation, as depicted in Figure 1D and Figure 2A–C [19]. We validated the selectivity of our cuff prototype on rat and mouse sciatic nerves, selectively activating the tibialis anterior (TA) and lateral gastrocnemius (LG) muscles through experimental validation and visual confirmation of fascicle orientation, highlighting the efficacy of our design.

## 2. Materials and Methods

### 2.1. Nerve Cuff Design and Fabrication

The primary objectives driving the development of this nerve cuff design included (1) ease of fabrication, (2) cost-effectiveness, (3) the incorporation of multiple channels for selectivity, (4) low impedance characteristics, (5) high repeatability, and (6) user-friendly attributes conducive to in vivo testing. We employed commercial FPC manufacturing techniques and widely available computer-aided design (CAD) software (EAGLE, Version 9.6.2) to achieve these goals. This method and software are accessible to all researchers, making it an ideal choice for our study.

Typically, FPCs feature a polyimide substrate with copper traces and an electroless immersion gold finish on exposed pads. We used an 8-electrode configuration in our prototype designs to demonstrate both circumferential and longitudinal selectivity. We accommodated differing nerve diameters through design variations fabricated together in a panel with four distinct size options (300, 500, 1150, and 1500 μm) and three attachment methods, as illustrated in Figure 2A,B. We ensured secure attachment of the cuff to the nerve without the need for sutures or additional holding mechanisms by implementing a looping mechanism akin to a zip-tie for two of the attachment designs and a folding pressure latch for the third optional design. The designs with the zip-tie featured a ratchet-like mechanism, allowing for even more size flexibility and a comfortable non-pressured fit (Figure 2C) [28]. To reduce electrode impedance and improve charge injection capacity, we coated the 100–500 μm diameter gold electrodes with poly(3,4-ethylene dioxythiophene) polystyrene sulfonate (PEDOT:PSS; PEDOTinks) post-fabrication. We compared manual application of the PEDOT:PSS polymer ink to nano-pipetting using nScrypt (Model: 150-3Dn-HP, nScrypt, Orlando, FL, USA) under a microscope and Fiji (ImageJ, Version 2.14.0/1.54f) imaging (Figure 2E). Both methods effectively coated the gold electrodes with a 1 μm layer of PEDOT:PSS. Since manual application was faster and less expensive, we used it for all subsequent tests in this study. This also aligned with our goal to develop an accessible approach that does not require specialized equipment. The end of the cuff assembly had a stiffener to form a standard FPC cable with 0.5 mm pitch. This connected to an FPC connector on the stimulator PCB.

### 2.2. In Vitro Electrode Characterization

We assessed the electrical characteristics of the PEDOT:PSS coating and its impact on impedance by subjecting the electrode to pulsed stimulation to measure the residual voltage ($V_{C}$) across time (*T*) during current injection (*I*), as given by the equation

(1) $$V_{C}=\frac{Q_{S}}{C_{I}}=\frac{I\cdot T}{C_{I}},$$

where $Q_{S}$ is the stimulation charge and *C* is the electrode’s interface capacitance. We monitored $V_{C}$ while injecting a 500 μA, 125 μs biphasic current pulse into a saline solution with a concentration of 0.90% w/v. We immersed a platinum wire in the saline solution with a surface area significantly larger than the electrodes and used it as the reference electrode in the experimental setup. The purpose was to ensure that the voltage on the electrode at the end of the first pulse did not exceed the water window for the electrode/electrolyte interface [29].

We measured the impedance of both the bare gold and PEDOT:PSS-coated electrodes (Figure 2E). We used a bench-top LCR meter (HP 4284A, Hewlett Packard, Hyogo, Japan) to conduct a frequency sweep, utilizing the lowest excitation voltage setting to mitigate the influence of electrode non-linearity. We immersed a platinum wire in the saline solution and used it as the reference electrode in the experimental setup, measuring the impedance of the electrode at each frequency.

### 2.3. In Vivo Testing

**Figure 3:**
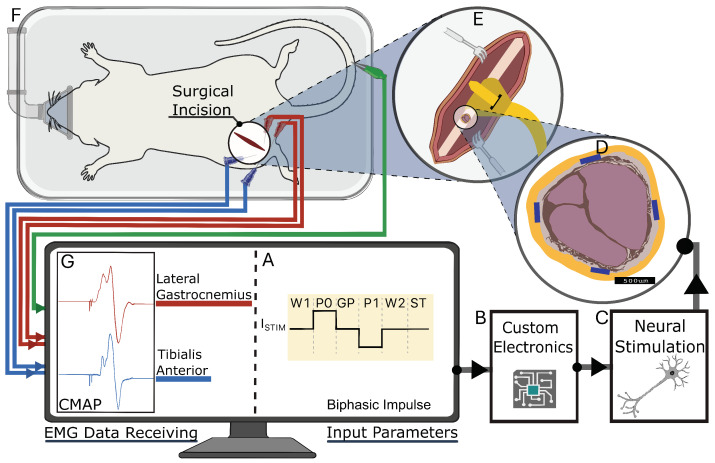
Experimental setup for neural stimulation in rodent model. (**A**) Biphasic impulse waveform with timing and characteristics of stimulation pulse, corresponding to stimulation chip design. (**B**,**C**) Wires connect custom electronics to peripheral nerve. (**D**) Cross-sectional view of sciatic nerve, highlighting radially placed electrodes in nerve cuff. (**E**) Cuff placement around sciatic nerve proximal to branches. (**F**) Subject positioned on surgical platform with surgical incision site and EMG electrode placement shown. (**G**) Computer monitor displaying Compound muscle action potentials (CMAPs) recorded from lateral gastrocnemius (red trace) and tibialis anterior (blue trace) muscles in response to neural stimulation.

All animal procedures were approved by the Institutional Animal Care and Use Committee (IACUC) and conducted in accordance with Boise State University’s PHS Assurance and AAALAC accreditation standards. Four adult male Sprague Dawley rats (400–480 g, age 6–9 mo; Idaho State University) and two adult female C57BL/6 mice (22–25 g, age 6–9 mo; Boise State University) were used for the experiments. The nerve cuff was surgically implanted around the sciatic nerve proximal to the branching of the tibial (T), peroneal (P), and sural (S) nerves that innervate the hindlimb muscles (Figure 3). The design and size of the nerve cuff used during in vivo stimulation were determined once the sciatic nerve was exposed and gauged for accessibility and diameter.

### 2.4. Surgical Procedure

We implanted the cuff on the left or right sciatic nerve of the subjects (Figure 3E,F). To anesthetize the subjects, we used vaporized isoflurane at a concentration of 3%, and then maintained anesthesia with 1.5–2.5% isoflurane in oxygen for the duration of the approximately 2 h procedure. The surgical site was shaved and prepared using standard aseptic techniques. Depending on the size of the subject, we made an incision approximately 1–3 cm in length, extending from the midpoint of the tibia to the midpoint of the femur, following the curvature of the knee joint. To access the sciatic nerve, we retracted the biceps femoris, exposing the nerve. Keeping the epineurium intact, we bluntly dissected the fascia securing the sciatic nerve to the underlying tissues. We then gently positioned the FPC nerve cuff under the nerve and secured it in its wrapped cuff position around the nerve. At the conclusion of the study, the animals were euthanized under anesthesia.

### 2.5. Stimulation Procedures

**Figure 4:**
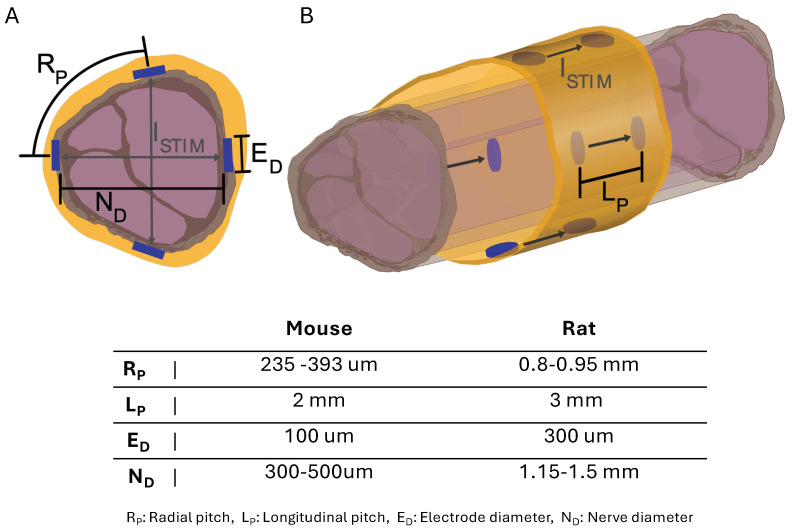
Diagram of sciatic nerve illustrating spatial stimulation ($I_{STIM}$) patterns at electrode points with electrode dimensions and positions for cuff sizes. (**A**) Nerve cross-section with electrode dimensions and radial $I_{STIM}$ patterns across nerve. (**B**) Longitudinal nerve view with electrode dimensions and $I_{STIM}$ patterns down nerve.

**Figure 5:**
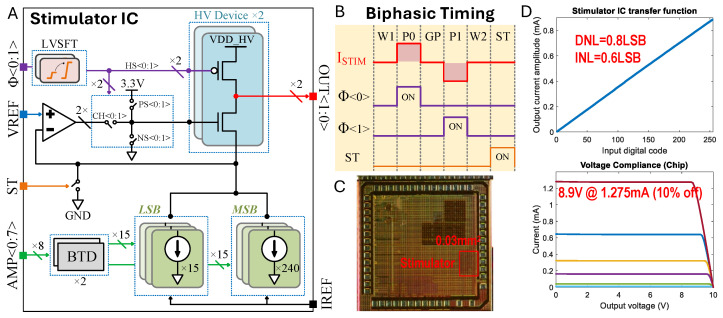
Custom stimulation chip design. (**A**) Stimulator integrated circuit (IC) architecture. (**B**) Biphasic ($I_{STIM}$) bipolar electrode stimulation timing diagram encoded by states (W1: Wait 1; P0: Phase 0; GP: Gap Phase; P1: Phase 1; W2: Wait 1 = 2; ST: Shorting Time). (**C**) Die micrograph. (**D**) Measured transfer function and compliance voltage, where the different colors indicate current settings of the stimulator.

We used a bipolar electrode configuration to demonstrate fascicle selectivity, pairing them either circumferentially or longitudinally (Figure 4). When surgically implanted, the electrode longitudinal pairs (1 to 4) were positioned counterclockwise from the cuff wrap junction (Figure 2C). A biphasic input current pulse with a pulse width of 150 μs and a 50 μs interphase delay between pulses was applied to the selected electrodes using a custom stimulation circuit (Figure 5). The current pulse amplitude was swept from 10 μA to 500 μA. The compound muscle action potentials (CMAPs) evoked by the stimulation were recorded through stainless steel electromyography (EMG) probes inserted into the tibialis anterior (TA) and lateral gastrocnemius (LG) muscles (Figure 3). We used an Analog Discovery 2 (Digilent) to differentially record the EMG signals at a sample of 500 kS/s. The recording was triggered by the stimulation control signal that was also generated by the Analog Discovery 2. To compute the selectivity index from the CMAP data, we used the equation

(2) $$SI_{1}=EMG_{1}-EMG_{2},$$

where EMG_1_ is the normalized standard deviation of the target muscle and EMG_2_ is the normalized standard deviation of the off-target muscle. We compared SI using Equation (2) to the more common SI calculation, given by the equation

(3) $$SI_{2}=\frac{EMG_{i}}{\sum_{i=1}^{N}EMG_{i}},$$

where *N* is the number of muscles assessed [30,31,32,33,34].

### 2.6. Stimulation Chip Design

We used a custom stimulation integrated circuit (IC) that we had previously developed [35]. Note that this specific system is not essential to use the proposed nerve cuffs. Figure 5A illustrates the stimulator IC architecture, the biphasic stimulation timing diagram (Figure 5B), the die micrograph (Figure 5C), and the measured transfer function and compliance voltage (Figure 5D). The stimulator IC was fabricated using a 180 nm HV CMOS process. We implemented an H-bridge with 10 V devices to enable biphasic current flow into a regulated cascode current sink. The amplifier of the regulated cascode was a folded-cascode OTA, which amplifies the output impedance by clamping the source node of the HV NMOS to VREF (250 mV). Consequently, we constructed an 8-bit current sink using core devices of 1.8 V. The current sink was designed with two 4-bit thermometer-encoded MSB and LSB blocks to balance layout complexity and linearity. Each unit reference within the LSB carries a nominal 5 μA, adjustable by IREF, resulting in a total current range of 0 to 1275 μA with a 5 μA step. Most control signals operate at the core voltage, but some control logic driving HV devices requires level shifting. We represented some correlated control signals (HS, CH, PS, NS) with the abstract signal ϕ<0:1>, where HS controls the HV PMOS, CH selects the channel for OTA and NS, and PS resets the HV NMOS. The ST signal can indirectly short both electrode outputs OUT<0:1> to ground for charge balance.

Figure 5B presents a digital timing diagram for biphasic stimulation. Biphasic stimulation mode includes six states: W1, P0, GP, P1, W2, and ST. Each state has a timing resolution of 2 μs and is set by a script. The wait and gap states (W1, W2, and GP) disable current flow, while P0 and P1 represent the two phases of biphasic stimulation, and the ST state shorts the electrodes to ground to ensure safe operation over multiple cycles. The experimental setup diagram (Figure 3) shows the overall stimulation architecture. Users can specify the stimulation parameters, including amplitudes and duration, via an Analog Discovery 2 Waveforms interface (Digilent). The PC then sends commands to the Analog Discovery 2, which directly controls the stimulator chip’s logic function. The on-board power management unit generates all necessary voltage supplies and references for the peripheral components.

The stimulator portion of the die measures 0.03 mm^2^ (Figure 5C). We measured the maximal differential non-linearity (DNL) and integral non-linearity (INL) to be 0.8LSB and 0.6LSB, respectively, by assessing the transfer function (Figure 5D). Voltage sweeps from 0 to 10 V under different load conditions were performed to characterize the voltage compliance specification. At a maximum load current of 1.275 mA, our measurements show a 10% decrease from nominal at 8.9 V with a VDDH supply of 10 V. Under nominal static conditions, 2 OTAs consume 30 μW (15 μW each). The reference current is adjustable from 1 to 10 μA if a finer resolution or a larger range is needed. With a unit reference current of LSB = 5 μA, the IDAC consumes around 60 μW. In total, the stimulator chip consumes approximately 90 μW.

### 2.7. Micro-Computed Tomography (μCT)

Following the stimulation procedure, we euthanized the subjects and marked each nerve for proximal and rotational orientation to the cuff during the stimulation. We then excised the nerves (≈1 cm) from the hind limb for fascicle imaging using μCT (SkyScan 1172). To perform imaging, we fixed the tissues in neutral buffered formalin (10% NBF, Sigma Aldrich HT501128) for 12 h and then submerged them in 1% Lugol’s solution (Sigma Aldrich L6141) for 24 h [36,37]. We then removed the tissues from the solution, blotted them dry to remove any excess Lugol’s solution, wrapped them tightly in cling film and medical gauze, and placed them in a 1 mL centrifuge tube filled with saline to avoid shrinkage of the nerve tissue. We conducted scans over a total length of 7 mm down the nerve at a voltage of 72 kV, a 140 μA current, and 10 W of power, as parameterized for stained neuronal soft tissues. We compared the fascicle size and location from scan slices to the fascicle predictions of location based on the EMG recruitment curve data.

## 3. Results

### 3.1. In Vitro Characterization

**Figure 6:**
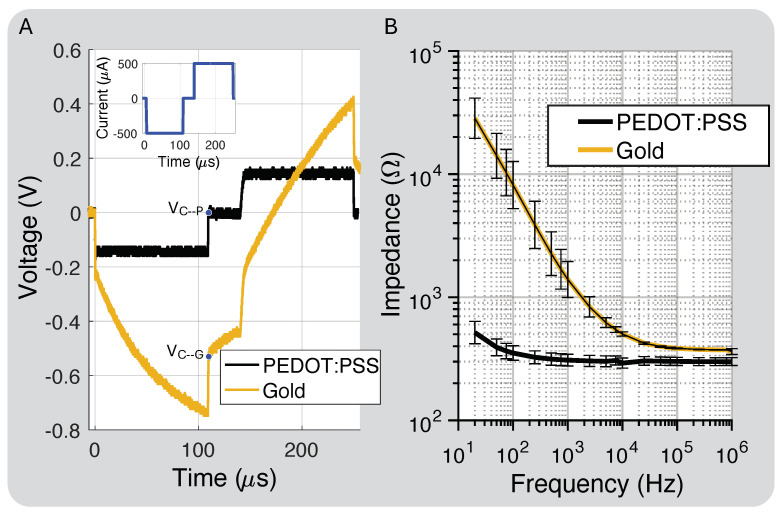
(**A**) Charge injection comparison for gold electrodes prior to and post PEDOT:PSS coating and residual electrode voltages ($V_{C-P}$ and $V_{C-G}$). (**B**) Impedance of electrodes coated with PEDOT:PSS compared to bare Au (standard deviation bars calculated for eight electrodes).

Figure 2D shows the polyimide cross-section layer thickness. The total thickness was 75 μm, and the metal traces and electrode layers were 17 μm with a 19 μm center division layer and 11 μm coverlay layer for the top and bottom layered designs. Figure 6A shows the PEDOT:PSS discharge capability compared to gold, close to our protocol’s highest intensity, which is well below the maximum charge injection capacity of the electrodes (1–10 mC/cm^2^) [38]. At this stimulation intensity, $V_{C-G}\approx$−0.54 V and $V_{C-P}\approx$ 0 V. This was expected because PEDOT:PSS should hold 75× more charge than gold based on the interface capacitance improvement.

Figure 6B shows the measured impedance for the bare gold and PEDOT:PSS-coated electrodes. PEDOT:PSS increased the interface capacitance by about 75×, which we attribute to both the interface enhancement of PEDOT:PSS and a slight increase in the electrode’s geometric surface area from the coating process [39]. The electrode spreading resistance, which dominates the impedance at higher frequencies, decreased by 20% due to the slight increase in surface area after the PEDOT:PSS coating.

### 3.2. Spatially Selective Nerve Stimulation

**Figure 7:**
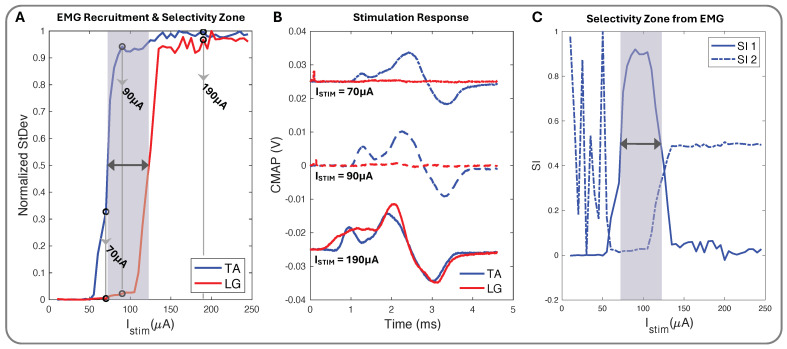
(**A**) Normalized stimulation recruitment curves for TA and LG with highlighted selectivity zone and three pinpointed current ($I_{STIM}$) amplitudes. (**B**) TA and LG CMAP EMG response curves for 70 (+0.025 V), 90, and 190 (−0.025 V) $I_{STIM}$ (μA). (**C**) SI_1_ (Equation (2)), SI_2_ (Equation (3)), and shadowed selectivity zone corresponding to (**A**) EMG recruitment data.

We used biphasic current stimulation with bipolar electrode pairs on the device to demonstrate the cuff’s ability to evoke fascicle-specific recruitment (Figure 4 and Figure 5). In the proximal sciatic nerve, we selectively stimulated the peroneal (P) and tibial (T) nerve fascicles and verified selectivity through the exclusive activation of the muscle groups those fascicles innervate. We recorded differential EMG data from the TA and LG muscles simultaneously for all stimulation configurations. We plotted normalized CMAP standard deviation values against the current stimulation amplitude to create the muscle group recruitment curves and a visual selectivity zone between the muscles’ activation, with the threshold value set to the normalized value of 0.5 (Figure 7A). The CMAPs at select current values for both TA and LG were easily observed to increase in peak-to-peak amplitude as the current increased (Figure 7B), creating the EMG recruitment curve from the CMAPs standard deviation and further illustrating the selectivity zone and its relationship to SI (Figure 7C).

**Figure 8:**
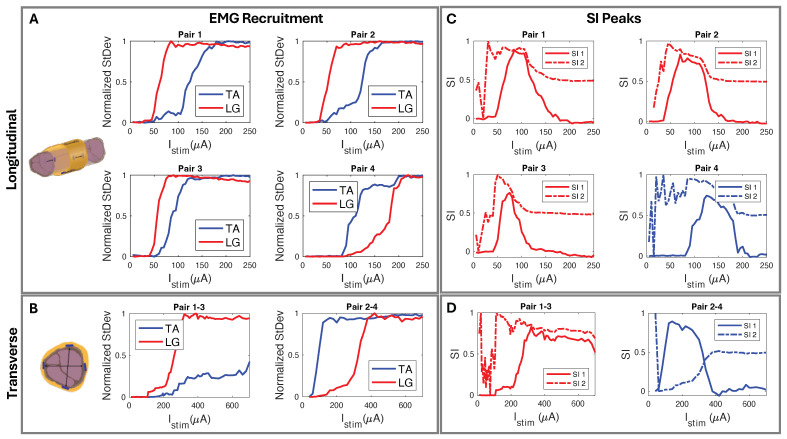
(**A**) Longitudinal and (**B**) transverse stimulation EMG recruitment curves from FPC cuff interfaced biphasic current injection. (**C**) SI corresponding to (**A**) longitudinal paired electrode EMG recruitment where, using SI_1_ Equation (2), Pairs 1–3 show selectivity for LG and Pair 4 selectivity for TA. (**D**) SI corresponding to (**B**) radial paired electrode EMG recruitment where, using SI_1_ Equation (2), Pairs 1–3 show LG selectivity and Pairs 2–4 show TA selectivity.

We used circumferential electrode pairings for transverse stimulation (Figure 4A). In one example subject shown in Figure 8, stimulating electrodes 2–4 transversely elicited high selectivity for TA. Conversely, stimulating electrodes 1–3 showed high selectivity for LG activation (Figure 8D). Each subject responded similarly, with slight differences due to anatomical variations and inevitable differences during cuff implantation. The longitudinal stimulation electrode pairs showed similar correlations in the subjects (Figure 8C). In the example subject data, the stimulation of electrode Pairs 1, 2, and 3 (Figure 4B and Figure 8C) often resulted in the LG muscle saturating more quickly than the TA. Conversely, the fourth pair showed a faster response in the TA.

**Figure 9:**
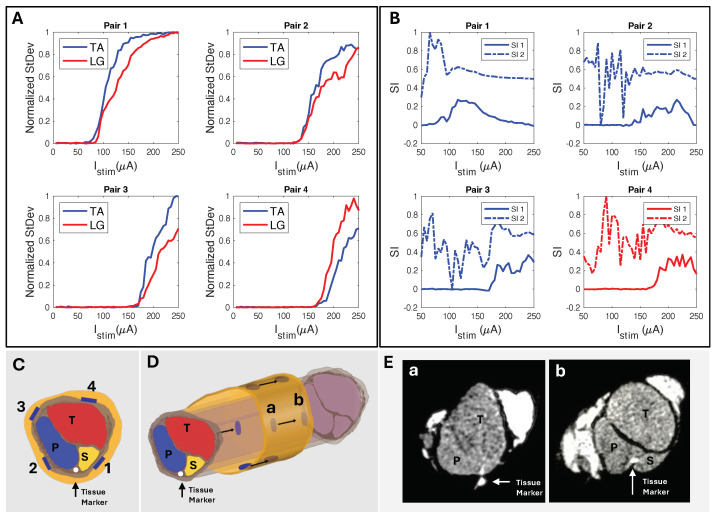
(**A**) Standard deviation of CMAP EMG response to longitudinally paired electrode current injection sites and their corresponding (**B**) SI responses (Rat N = 4). (**C**) Predicted electrode orientation based on SI data. (**D**) Longitudinal view showing locations **a** and **b** for cross−sectional slices. (**E**) μCT of sciatic nerve sectioned at longitudinal locations (**a**,**b**) (Rat N = 4), confirming SI data and fascicle−to−electrode orientations.

The peak SI data (Table 2), calculated with Equation (2), occurred in the “selectivity zone” (Figure 7A,C) of current amplitude. The zone width of current was determined for peaks above 0.5. We further confirmed the selectivity data through μCT imaging. Using SI data from the corresponding subject (Figure 9A), we predicted electrode spatial locations and proximity to fascicles (Figure 9B,C). The cuff tissue marker indicated the gap point between electrodes 1 and 4 during μCT, allowing us to confirm the fascicle size and spatial locations in proximity to the marker of electrode stimulation points (Figure 9D). This confirmed our predictive analysis of the SI data for that subject and further verified the spatially selective stimulation capability of the cuff.

## 4. Discussion

### 4.1. Nerve Cuff Design and Fabrication

The FPC market offers a diverse range of manufacturers, allowing us to select the most suitable option for our project. We considered key factors such as production time (ranging from 24 h to 4 weeks), cost (dependent on design complexity), and resolution capabilities (from 25 to 200 μm). The diversity of options provided us with the flexibility to develop multiple designs concurrently and to modify designs with minimal time loss and expense. Of the three designs that we created and tested, the zip-tie design provided the best ease of use and handling at millimeter sizing. The other two designs, while functional, were more difficult to implant in vivo.

The evaluation and measurement of our FPC thickness of 75 μm was less than the manufacturing specification of 100 μm. However, the thinner substrate was advantageous in that the curvature and bending radius allowed the cuff to be modified to even smaller nerve diameters. Based on $\epsilon =\frac{h}{2r}$, where ϵ is the bending strain, h is the thickness of the FPC, and r is the smallest nerve diameter for the bending radius [40,41], the predicted strain for our cuff design decreased by 8% for a polyimide thickness of 75 μm compared to 100 μm. By using a thinner substrate in our cuff fabrication, the smaller-diameter cuffs became easier to achieve and use. In addition to the advantage of flexibility for small bending radii, polyimide has been used for neural interface applications due to its biocompatibility and chemical resistance [41,42]. While polyimide as a material has been proven to be effective in numerous studies for neuronal interfaces [42,43,44,45], discerning the exact types of materials used in commercial FPC fabrication can be difficult. FPC polyimide is often referred to as Kapton, a thin-film thermosetting polyimide used primarily for FPCs. One important consideration for chronic neural cuffs is the water absorption level of the material. Considering future applications, we subjected the polyimide to a 24 h water absorption test in accordance with ASTM standard D570 [46]. Polyimide’s water absorption can range between 0.4 and 4% [47,48,49,50,51,52]. We measured a water absorption of 0.66%. Although current research trends are to develop softer cuffs that match the tensile strength of nerves [1,53], our proposed method enables rapid design iteration with high spatial selectivity at a fraction of the cost and time of microfabrication approaches.

### 4.2. In Vitro Electrode Characterization

The PEDOT:PSS coating led to lower impedance and a higher charge injection capacity compared to bare gold electrodes. In addition to increasing the total charge injection capacity [54,55], the lower-impedance PEDOT:PSS electrodes required less headroom voltage to inject the same amount of stimulation charge (Figure 6B). This is critical for low-power applications [35,56]. From a safety perspective, the PEDOT:PSS coating helps ensure the residual electrode voltage, $V_{C}$, remains well within the electrochemical water window (+0.6 V to −0.9 V) [39,57]. Furthermore, PEDOT:PSS’s superior charge and discharge capability is important for closed-loop neuromodulation where recording starts immediately after stimulation (Figure 2A) [58,59] and has been shown to be a better neural recording electrode material with higher SNRs [60].

One concern about the hand application of PEDOT:PSS is potential variability between electrodes. We can quantify this by comparing the electrode impedance at high frequency, where it is dominated by the spreading resistance. For small voltages, the impedance magnitude of the electrode is roughly

(4) $$|Z_{elec}\left(\omega \right)|\approx |R_{S}+1/j\omega C_{I}|,$$

where $R_{S}$ is the spread resistance as a function of the radius of the electrode ($R_{S}$ = $\frac{\rho }{4r}$) [61]. The $R_{S}$ for gold electrodes was 377 +/− 3 Ω and the $R_{S}$ for PEDOT:PSS was 300 +/− 24 Ω. These standard deviations correspond to effective radius differences of 0.8 and 8%, respectively. Hand application has comparable variation to the nScrypt application process, which is minimal. Considering this processing step takes only a few minutes for an entire panel of nerve cuffs, hand application is the preferred method. While PEDOT:PSS may delaminate over time in solution or in vivo [57,62], we did not explore this because the coating did not delaminate during our experiments.

### 4.3. In Vivo Selectivity Index

Conventionally, the selectivity index (SI) is computed by dividing the EMG energy of the target muscle by the total EMG energy of all recorded muscles (Equation (3)) [30,31,32,33]. This is most effective when three or more muscles are simultaneously being evaluated. However, this can make a small level of activation (e.g., 0.1) in the target muscle appear highly selective (e.g., 1) if no other muscles are activated above the noise floor. This is evident in Figure 7 and Figure 8, where SI_2_ consistently showed multiple instances of absolute selectivity (SI = 1) before appreciable activation of the target muscle EMG. Our method of computing SI (Equation (2)) was developed specifically for this study. We computed SI by taking the difference between the normalized EMG activation levels, where a selectivity of 1 indicates that the target muscle was fully recruited without any off-target muscle recruitment. By calculating SI in this way, selective activation was better correlated with physiological responses than the conventional SI calculation (Equation (3)).

### 4.4. Spatially Selective Nerve Stimulation

We developed a cuff that is easy to manufacture and is capable of selective stimulation. We used it to selectively stimulate the peroneal (P) and tibial (T) nerve fascicles and activate the corresponding muscle groups, the anterior tibialis (TA) and the lateral gastrocnemius (LG). By stimulating with longitudinal patterns, we selectively stimulated both the TA and LG muscle groups, with LG selectivity observed in at least one electrode pair in five of six subjects (Table 1, Figure 8C). Since the P fascicle that innervates the TA was usually more responsive to stimulation, we observed that the TA recruited more quickly than the LG in most subjects. This is likely due to the thickness of the perineurium and the diameter of the fascicle, where smaller fascicles with a thinner perineurium have lower activation thresholds [63]. The experiments demonstrating LG selectivity was likely due to closer proximity to the T nerve bundle. Transverse stimulation across the nerve cross-section showed similar patterns (Figure 8D) to longitudinal stimulation (Figure 8C), allowing us to selectively stimulate both TA and LG. Our goal was not to place the cuff in the same position for every experiment, but to establish spatial selectivity through iterative multi-channel nerve stimulation with our inexpensive and easy-to-manufacture approach. We adjusted each cuff’s tightness to the subject’s nerve diameter, and electrode positioning relative to fascicles was determined post hoc from EMG recruitment data. In a subject that demonstrated poor selectivity (SI < 0.5), we performed μCT to confirm that the position of the electrodes aligned with the predicted fascicle locations (Table 2). We concluded that the poor selectivity was likely due to the thick connective tissues that prevented effective current delivery to the fascicles (Figure 9E).

Compared to rats, mice’s nerves are smaller and require a smaller current for muscle recruitment, which means spatial selectivity is more challenging. However, we successfully demonstrated selectivity for LG and TA in one subject and selectivity for TA in another. To our knowledge, this is the first demonstration of spatial selectivity with low-cost epineural electrodes in both rats and mice.

## 5. Conclusions

We introduced a straightforward and accessible method for creating multi-channel epineural nerve cuffs using commercial flexible printed circuit (FPC) technology. This method does not require any specialized equipment for widespread adoption. These cuffs offer high spatial resolution (50 μm) and customizable parameters, addressing the need for cost-effective and rapidly prototyped designs in selective neuromodulation acute research. The PEDOT:PSS coating on the electrodes significantly improved the charge injection capacity to ensure stimulation remained within acceptable safety limits. Using our nerve cuff, we demonstrated selective activation of the tibialis anterior (TA) and lateral gastrocnemius (LG) muscles in both rats and mice. The efficacy of our design was further validated through micro-computed tomography (μCT) imaging, which confirmed the spatial relationships between cuff electrodes and nerve fascicles.

This approach overcomes several limitations of existing fabrication methods, including high costs, time-intensive production, and challenging design iterations. By enabling the rapid and cost-efficient prototyping of multi-channel nerve cuffs, our method paves the way for more extensive exploration of selective neuromodulation therapies in acute studies. Future work will focus on exploring applications in treating various disorders responsive to neuromodulation. Ultimately, this research contributes to the advancement of more precise and effective neuromodulation treatments, potentially improving patient outcomes and quality of life.

## Figures and Tables

**Table 1:** Comparison of published and commercial neuromodulation cuff fabrication methods.

	Manual	Additive	Microfab	Laser	Commercial
**Minimum**	>1 mm	>30 μm	>50 nm	>200 μm	>100 μm
**Electrode Size/Pitch**		>250 μm			>150 μm
**Channel No.**	2	2–4	8	3	2–8
	4				2
**No. Cuffs**	1	1	Wafer (1+)	1	NA *
**per Fabrication**					
**Equipment** **Required**	Microscope, >34 Assembly Items	Inkjet Printer Micro-extrusion	Cleanroom, Photolithographic Equipment	CO_2_	NA *
**Electrode Material**	Pt	PEDOT:PSS	TIN, Gold	Silicone/	Stainless Steel, Pt, PtIr
	PtIr		PEDOT:PSS	Carbon Black	PtIr
**NRE Cost**	High	Moderate	High	Moderate	High
**Modification**	Moderate	Moderate	Difficult	Moderate	Difficult
**Capability**	to Difficult				
**Citation**	[23]	[21]	[1]	[25]	[26]
	[24]	[22]	[20]		[27]

* Proprietary information unavailable.

**Table 2:** Peak SI data and corresponding stimulation zone width for all subjects’ longitudinal and transverse electrode pairings.

	Longitudinal						Transverse					
	**Peak** ${\mathrm{SI}}_{1}$**(Equation (2)**)		**Peak** ${\mathrm{SI}}_{2}$ **(Equation (3))**		${\mathrm{SI}}_{1}$ **Zone Width (**μ**A)**		**Peak** ${\mathrm{SI}}_{1}$ **(Equation (2))**		**Peak** ${\mathrm{SI}}_{2}$ **(Equation (3))**		${\mathrm{SI}}_{1}$ **Zone Width (**μ**A)**	
**Rat**	**TA**	**LG**	**TA**	**LG**	**TA**	**LG**	**TA**	**LG**	**TA**	**LG**	**TA**	**LG**
**1**	0.41 *	-	0.69	-	*	*	0.60	0.90	0.76	0.93	400	220
**2**	0.99	0.75	1	1	55	25	0.89	0.83	1	1	225	610
**3**	0.74	0.88	1	1	55	67	-	0.81	-	1	*	290
**4**	0.37 *	0.37 *	1	1	*	*	0.44 *	-	1	-	*	*
**Mouse **												
**1**	0.79	0.76	1	1	27	18	0.45 *	0.96	0.73	0.98	*	76
**2**	0.66	0.45 *	1	1	70	*	-	-	-	-	*	*

‘-’ SI peak not observed. * SI peak did not reach threshold (0.5).

## Data Availability

The raw data supporting the conclusions of this article will be made available by the authors on request.
